# High b-value diffusion-weighted imaging in progressive multifocal leukoencephalopathy in HIV patients

**DOI:** 10.1007/s00330-017-4761-8

**Published:** 2017-02-06

**Authors:** Claudia Godi, Enrico De Vita, Enrico Tombetti, Indran Davagnanam, Lewis Haddow, Hans Rolf Jäger

**Affiliations:** 1 Neuroradiology Department, San Raffaele Scientific Institute, Milan, Italy; 2 Lysholm Department of Neuroradiology, The National Hospital for Neurology and Neurosurgery, Queen Square, London, UK; 3 Neuroradiological Academic Unit, Department of Brain Repair and Rehabilitation, UCL Institute of Neurology, Queen Square, London, UK; 4 Vita-Salute San Raffaele University, Milan, Italy; 5 Centre for Sexual Health and HIV Research, Research Department of Infection and Population Health, University College London, London, UK; 6 Centre of Medical Imaging, University College Hospital, London, UK

**Keywords:** Progressive multifocal leukoencephalopathy, Human immunodeficiency virus, Diffusion magnetic resonance imaging, Diffusion MRI, Diffusion-weighted MRI

## Abstract

**Objectives:**

An ill-defined hyperintense edge and hypointense core on diffusion-weighted imaging (DWI) is typical of progressive multifocal leukoencephalopathy (PML). We aimed to investigate whether a b-value of 3,000 s/mm^2^ (b3000) can improve visualisation of PML, or provide different structural information compared to 1,000 s/mm^2^ (b1000).

**Methods:**

We retrospectively identified HIV-positive patients with confirmed PML studied under a clinical protocol including both b1000 and b3000 DWI. The rim and core of each PML lesion and normal-appearing white matter (NAWM) were outlined on trace-weighted DWI. Signal intensities, apparent diffusion coefficient (ADC) values and volumes were measured and compared between b1000 and b3000.

**Results:**

Nine lesions from seven patients were analysed. The rim and core were better visualised on b3000, with higher signal of the rim and lower signal of the core compared to NAWM. The hyperintense rim had non-restricted average ADCs, but included foci of low ADC on both b3000 and b1000. Despite similar total lesion volumes, b3000 displayed significantly larger core and smaller rim volumes than b1000.

**Conclusion:**

b3000 improves visualisation of this important PML hallmark. Moreover, b3000 partly reclassifies tissue from rim into core, and might provide potentially more accurate biomarkers of PML activity and prognosis.

**Key Points:**

• *B3000 improves contrast resolution between lesion rim, core and normal-appearing white matter*.

• *B3000 improves identification of the typical rim-and-core pattern of PML lesions*.

• *B3000 and b1000 similarly identify lesions, but b3000 results in smaller rims and larger cores*.

• *B3000 excludes some high diffusion components from rim, reclassifying them into core*.

• *B3000 DWI may provide more precise PML biomarkers of disease activity and tissue damage*.

**Electronic supplementary material:**

The online version of this article (doi:10.1007/s00330-017-4761-8) contains supplementary material, which is available to authorized users.

## Introduction

Progressive multifocal leukoencephalopathy (PML) is a rare, potentially life-threatening demyelinating disease of the brain white matter [1] caused by JC virus (JCV) [2, 3] in immunocompromised hosts.

PML is a typical complication of patients with HIV-infection (HIV^+^) [4], but over the last decade has been increasingly described in the setting of immunosuppressive regimens, including natalizumab in patients with multiple sclerosis (MS) [5–8].

An early diagnosis of PML is crucial to optimise clinical management of patients (e.g. by potentiating combined anti-retroviral therapy (cART) in HIV+ patients [9], or reducing the intensity of iatrogenic immunosuppression) and to improve long-term outcomes [10].

When typical pathological findings are present [11], histology allows a definite diagnosis of PML. However, brain biopsy has many drawbacks, including low sensitivity due to sampling errors and possible complications from the invasive procedure. Last but not least, it entails a previous clinical suspicion of PML.

All these pitfalls highlight the importance of alternative and less invasive diagnostic clues. Recently, the American Academy of Neurology (AAN) Neuro-infectious Disease Section reviewed the diagnostic criteria for PML and established that PML diagnosis can be made with different degrees of certainty (possible, probable and definite) upon a combination of typical clinical imaging findings, and evidence of JC virus in the cerebrospinal fluid (CSF) [11].

According to this consensus statement, imaging plays an important role in suggesting or confirming PML diagnosis. Compatible imaging findings are white matter lesions that often start in the subcortical regions with involvement of the U-fibres, and then move into the deeper white matter of the centrum semiovale and eventually evolve into full blown, largely confluent T2 hyperintense and T1 hypointense non-enhancing lesions. Infratentorially the PML lesions typically involve the middle cerebellar peduncles [12–14]. In the setting of HIV infection, other conditions like HIV encephalopathy can affect white matter and represent a diagnostic challenge [12, 14, 15]. An increased MRI accuracy for PML would thus be highly desirable in order to recognise PML lesions in case of unspecific findings on T1- and T2-weighted images. This may in the end reduce the necessity of brain biopsy or even of a JCV-positive CSF for PML diagnosis.

Of note, PML lesions can show an ill-defined rim of high signal intensity at the advancing edge and a hypointense core on diffusion-weighted imaging (DWI) [16–18]. This PML feature is peculiar and differs from the DWI findings observed in other demyelinating and non-demyelinating diseases [19–21]. Histological-radiological comparisons demonstrated that DWI peripheral bright rim and hypointense core were histologically related to swollen JCV-infected oligodendrocytes at the advancing edge of the lesion [22, 23] surrounding a central area [24] of demyelination with axonal loss. Taking these findings into account, this unique rim and the core pattern displayed on DWI could be of particular clinical interest, as the presence of swollen oligodendrocytes at the hyperintense edge is believed to represent ongoing demyelination, and possibly a marker of active disease [16], whereas the hypointense core corresponding to axonal loss and increased extracellular space may identify irreversibly damaged brain tissue [25].

The use of b-values higher than 1,000 s/mm^2^ increases the diffusion-weighting power of DWI [26–28]. Based on previous evidence from a single patient [29], we hypothesised that DWI with a b-value of 3,000 s/mm^2^ (b3000) can improve the visualisation of the rim and core of PML lesions compared to the standard DWI with a b-value of 1,000 s/mm^2^ (b1000). In this retrospective study we explore whether b3000 DWI provides a greater contrast of PML rim to normal-appearing white matter (NAWM) and a different definition of the rim and the core compared to b1000. Finally, we aimed to estimate the contribution of T2 and diffusion effects to the DWI “hyperintense rim” phenomenon by measuring the ADC values of rim and core with both b-values.

## Materials and methods

### Study design and criteria

Imaging data from HIV-positive patients referred to UCLH (University College London Hospitals) for brain MRI between 2004 and 2011 were retrospectively reviewed. Inclusion criteria were: a definite diagnosis of PML in accordance with the Consensus Statement from the AAN Neuro-infectious Disease Section [11] (clinical and imaging findings consistent with PML, confirmed by positive nucleic acid amplification of JCV-specific DNS in the CSF), positive HIV-antibody status, and at least one brain MRI study performed using b1000 and b3000 DWI sequences before the start of cART. Patients with a PML ‘non-definite’ diagnosis (e.g. lack of CSF virological confirmation) or with HIV-PML already under treatment were excluded.

### Imaging data

Imaging was performed on 1.5-T systems (MAGNETOM® Symphony and Avanto, Siemens, Erlangen, Germany) and included standard brain imaging with sagittal and coronal spin echo T1-weighted images, axial fast spin echo T2-weighted images and coronal FLAIR images, as well as axial and coronal spin echo T1-weighted images after gadolinium administration. Axial trace-weighted DWI images with b-values of both 1,000 and 3,000 s/mm^2^ (TR range 3,200–3,900 and 3,200–4,400 ms for b1000 and b3000, respectively; TE range 81–97 and 113–133 ms for b1000 and b3000, respectively; matrix 128 × 128, field of view (FOV) 230 × 230 mm^2^, slice thickness 5 mm, gap 1.5 mm) were acquired alongside b = 0 s/mm^2^ images with corresponding parameters. The ADC maps generated automatically by the manufacturer’s software were used.

### Regions of interest

The rim and core of each PML lesion were identified and manually outlined on consecutive axial slices of both b1000 and b3000 trace-weighted DWI, using FSLView with standardised window settings (0–200 for b1000 DWI, 0–120 for b3000 DWI). All regions of interest (ROIs) were set in consensus by two observers, initially drawn by a more junior reader (8 years experience in neuroradiology), then reviewed and confirmed by a more experienced reader (25 years experience in neuroradiology). The rim was identified as the bright edge of the lesion, and the core as the central hypointense area of each PML lesion. Rim and core were outlined on all the slices in which they were visible, then automatically combined to obtain rim and core volumes. Control ROIs were also placed on each slice on subcortical normal-appearing white matter (NAWM) in areas deemed to be unaffected on T2 and FLAIR images.

ROI signal intensities (SIs) were measured on b1000 and b3000 DWI; the same ROIs generated with DWI trace-weighted images were then superimposed on ADC maps to get the corresponding ADC values. DWI rim and core SI values were finally normalised to those of control ROIs to obtain relative signal intensity ratios (SIRs).

### Statistical methods

Statistical analyses were performed with GraphPad PRISM Software, version 6.0 (La Jolla, CA, USA). The Wilcoxon signed-rank test was used to compare values between b1000 and b3000 datasets. P-values < 0.05 were considered significant.

## Results

### Population

Seven patients with a definite diagnosis of PML (three females, four males, median age: 43 years, range: 19–66 years) fulfilled the inclusion criteria. Patients’ demographics and clinical details are provided in Table 1.

**Table 1:** Demographics and clinical features of progressive multifocal leukoencephalopathy (PML) patients (*n* = 7)

Patient	Gender	Age, y	Symptoms at diagnosis	Location of the PML lesion	Blood CD4^+^ lymphocytes (absolute count with %)^a^	Plasma HIV viral load^b^
#1	M	49	Unknown	1. Right parietal	140 (11.7%)	25,400
				2. Left occipito-parietal		
#2	M	31	Right hemiparesis	1. Left fronto-parietal	<10 (0%)	2,898,500
				2. Right frontal		
#3	F	19	Dysarthria, dysphagia, right-sided weakness	Left fronto-temporal	50 (8.4%)	1,800
#4	F	34	Left hemiparesis	Right fronto-parietal	70	50,000
#5	M	66	Progressive left hemiparesis	Right frontal	290 (16.1%)	290,000
#6	M	44	Progressive cerebellar syndrome	Left middle cerebellar peduncle	160 (14.5%)	425,300
#7	F	58	Dysphasia, right hemiparesis	Left frontal	390 (10.2%)	250,000

*M* male, *F* female

^a^ Cells/μl, normal range: 350–1,250

^**b**^ Copies/ml

Five patients had a single white matter lesion, and the remaining had two distinct lesions (patients #1 and #2). In total, nine PML lesions were analysed: every one apart from patient #6 was located in the supratentorial white matter. All the lesions were hypointense on T1-weighted, hyperintense on T2-weighted and FLAIR images, showed no contrast enhancement, and displayed the typical rim-core pattern with a peripheral rim of high signal and a central area of low intensity on DWI.

### The rim and core definition differs between b3000 and b1000 DWI

**Fig. 1:**
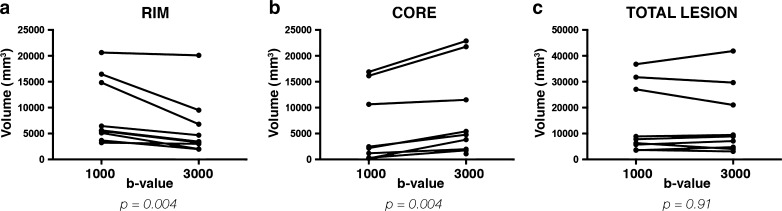
b1000 and b3000 volumes (in mm^3^) of the rim and core of all progressive multifocal leukoencephalopathy (PML) lesions are shown in panels **A** and **B**, respectively. Overall, the rim volumes on b3000 were lower, and the core volumes higher than their counterparts on b1000 diffusion-weighted imaging without any significant change in the total lesion volume being noted (**C**)

**Fig. 2:**
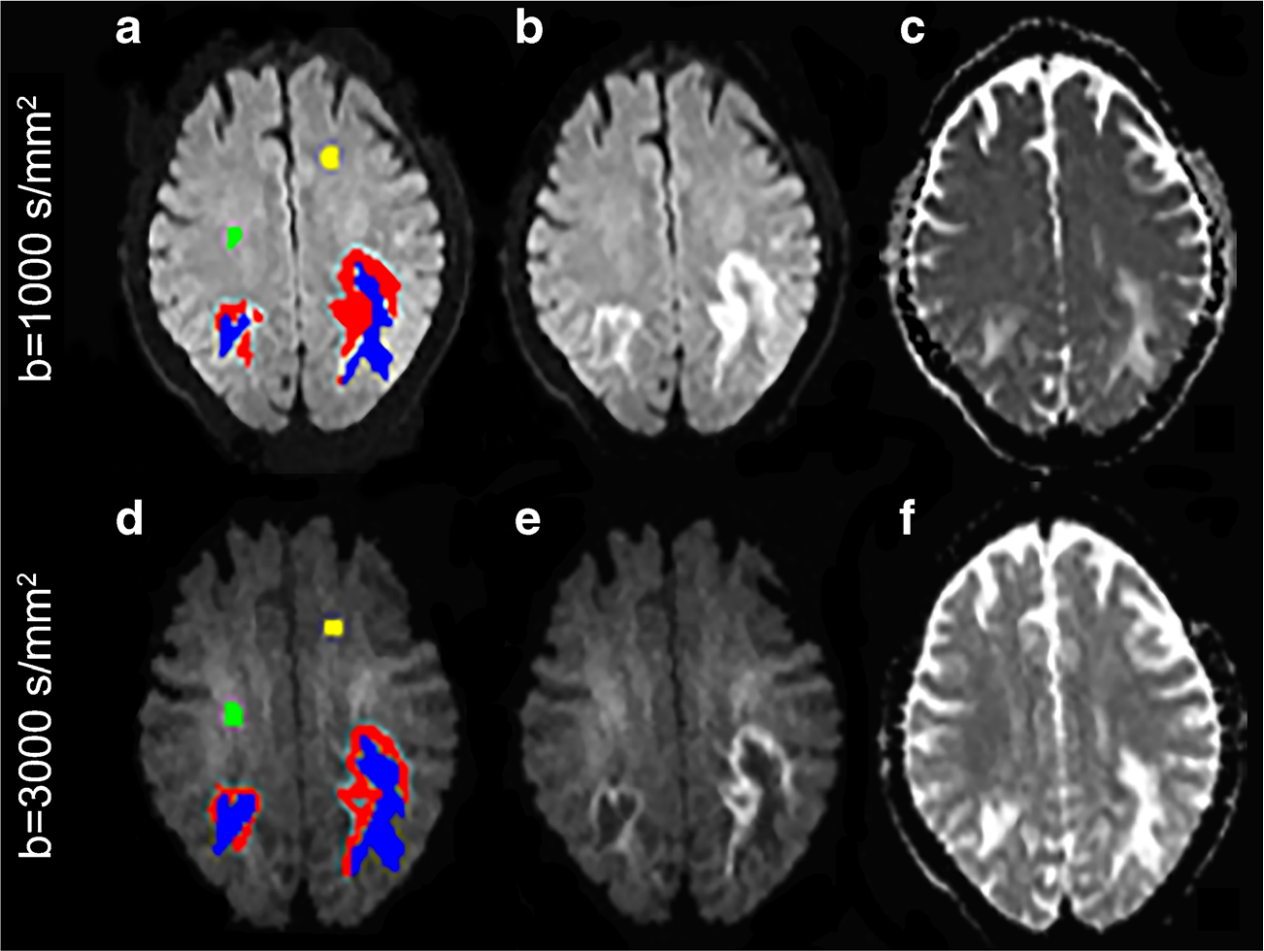
A representative patient is shown in images **A**–**F**. On b1000 (**B**) and b3000 (**E**) trace-weighted diffusion-weighted imaging, progressive multifocal leukoencephalopathy (PML) rim is outlined in red, PML core in blue, and normal-appearing white matter (NAWM) in yellow and green (**A**, **D**). Although similar lesion volumes were identified by the two b-values (sum of red and blue components), the rim in b1000 appeared thicker than in b3000 (**B**, **E**); conversely, the core in b3000 was more identifiable and larger than in b1000, not only on trace-weighted images (**B**, **E**) but also on apparent diffusion coefficient (ADC) maps (**C**, **F**)

A similar delineation of PML lesions was obtained by b1000 and b3000 DWI, with comparable total volumes (b1000 DWI: 7.81 cm^3^, interquartile range (IQR) 4.69–29.43, vs. b3000 DWI: 8.87 cm^3^, IQR 4.38–25.34; *p = 0.91*; Fig. 1C). However, the two b values significantly differed in characterising components of PML lesions: in particular, b3000 DWI provided significantly smaller rims than b1000 (3.44 cm^3^, IQR 2.51–8.16, vs. 5.62 cm^3^, IQR 4.40–15.66, *p = 0.004*; Fig. 1A) and significantly bigger cores (4.77 cm^3^, IQR 1.88–16.63 vs. 2.19 cm^3^, IQR 0.27–13.39, *p = 0.004*; Fig. 1B). Thus, b1000 and b3000 DWI similarly identified PML lesions, but resulted in a different characterisation of the lesion components, with a more stringent delineation of the rim with b3000 DWI (Fig. 2), and better delineation of the core on both trace-weighted images and ADC maps (Figs. 2E and F).

### The contrast between rim, core and NAWM is increased with b3000 DWI

As expected with high b-values, SIs of the lesion rim (SI_rim_), lesion core (SI_core_) and NAWM (SI_NAWM_) on b3000 were lower than on b1000 trace-weighted DWI (Table 2). SI_rim_ was the highest on both b1000 and b3000 trace-weighted DWI, followed by SI_NAWM_ and SI_core_, respectively. Of note, SI_core_ highly abated with b3000 DWI (Fig. 2).

**Table 2:** Signal intensity (SI) and apparent diffusion coefficient (ADC) values (×10^-7^ s/mm^2^) of progressive multifocal leukoencephalopathy (PML) rim, normal-appearing white matter (NAWM) and PML core from b1000 and b3000 diffusion-weighted imaging datasets

	b1000 SI	b3000 SI	b1000 ADC	b3000 ADC
Rim	172.8 (162.2–187.5)	69.0 (60.2–77.8)	91.9 (78.3–97.6)	51.9 (45.1–56.6)
NAWM	109.8 (97.6–114.3)	33.1 (32.4–35.4)	74.6 (72.9–77.9)	49.6 (46.6–51.2)
Core	95.8 (76.2–125.3)	22.4 (18.8–26.2)	163.0 (152.5–186.4)	100.2 (90.0–105.6)

Values are expressed as median and interquartile ranges (25–75 percentiles)

The contrast (expressed by the SIR) between the lesion rim and NAWM and between the lesion core and NAWM on b3000 DWI was higher than on b1000 DWI images (SIR: 2.02, IQR 1.80–2.28 vs. 1.64, IQR 1.49–1.71, *p = 0.004* and 0.68, IQR 0.57–0.79 vs. 0.98, IQR 0.71–1.17, *p = 0.023*, respectively). Moreover, b3000 DWI strongly highlighted the contrast between the rim and the core of PML lesions in comparison to b1000 DWI (SIR: 2.98, IQR 2.50–3.73 vs. 1.61 IQR 1.41–2.15, *p = 0.008*, Tables 2 and 3), with a particularly evident signal drop in the core of b3000 (Fig. 2E). These data clearly show that b3000 improves the contrast resolution between lesion rim, core and normal-appearing white matter, facilitating the recognition of the rim-and-core pattern, which is a hallmark of PML.

**Table 3:** Signal intensity ratios (SIRs) of b1000 and b3000 diffusion-weighting imaging datasets. Significant p-values (<0.05) are given in bold

	b1000 SIR^a^	b3000 SIR^a^	p-value^b^
Rim/NAWM	1.64 (1.49–1.71)	2.02 (1.80–2.28)	**0.004**
Core/NAWM	0.98 (0.71–1.17)	0.68 (0.57–0.79)	**0.023**
Rim/core	1.61 (1.41–2.15)	2.98 (2.50–3.73)	**0.008**

^a^ Values are expressed as median and interquartile ranges (25–75 percentiles)

^b^ Wilcoxon signed rank test

*NAWM* normal-appearing white matter

### DWI hyperintense rim consists of regions with different ADC values and b3000 excludes some high ADC areas from the rim

Table 2 shows the ADC values of the lesion rim, lesion core and NAWM on b1000 and b3000 DWI. As expected, b3000 resulted in lower ADC values and PML cores showed the highest ADC with both b1000 and b3000.

**Fig. 3:**
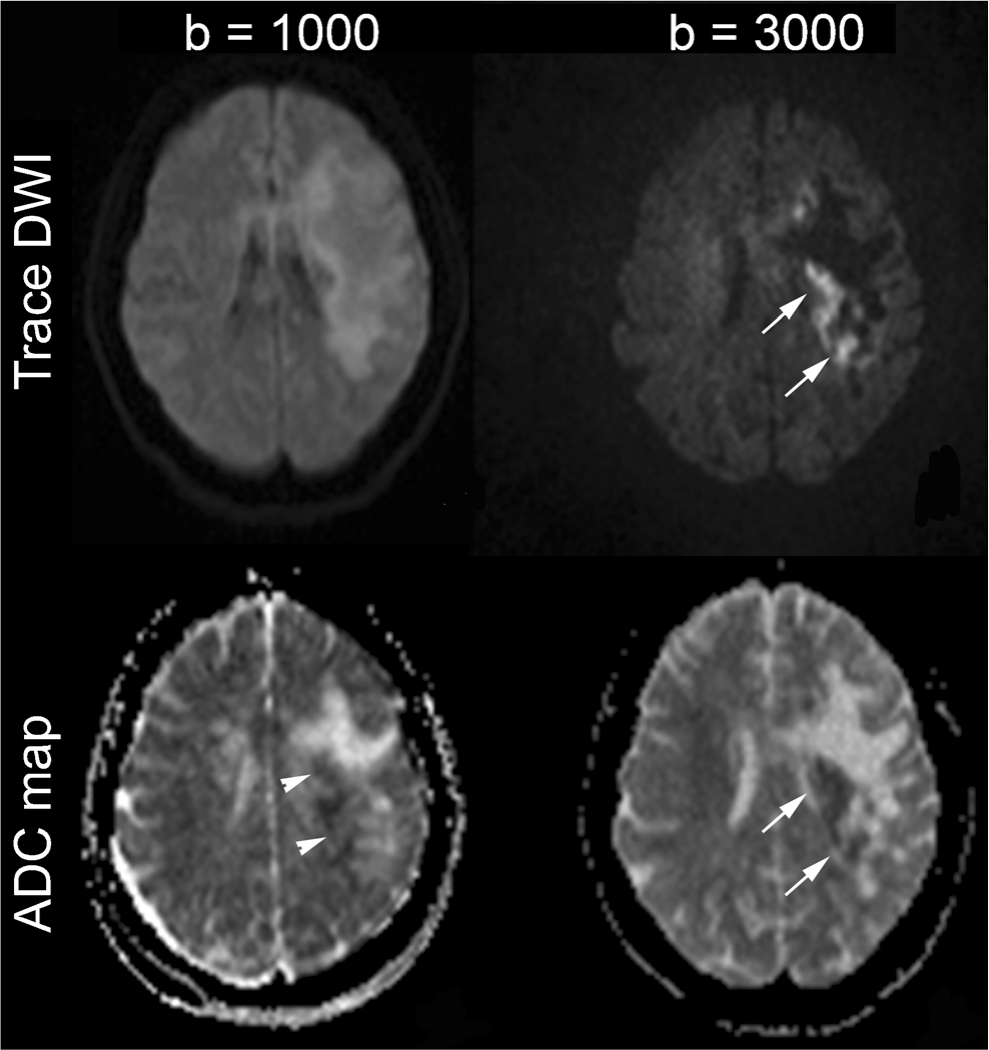
At b1000 the hyperintense progressive multifocal leukoencephalopathy (PML) rim is a heterogeneous region that corresponds to intermediate apparent diffusion coefficient (ADC) values on average, although some spots of low ADC values within the rim are observed (*arrowheads*). However the rim on b3000 diffusion-weighted imaging (DWI) contains a higher proportion of low ADC values (*arrows*), suggesting that the more stringent delineation of the lesion rim on b3000 results in exclusion of some areas with increased diffusion

On b1000 DWI, average ADC values of the lesion rim were slightly higher than those of the NAWM (*p = 0.022*): however, ADC values within the rim appeared to be heterogeneous, with definite foci of low ADC (Fig. 3, arrowheads).

We found all the spots with low ADC values identified on b1000 were included within the rim on b3000 DWI (Fig. 3, arrows), suggesting that the more stringent delineation of lesion rim on b3000 resulted in the exclusion of some areas with increased diffusion. Concordantly, average b3000 ADC values of the rim were similar to NAWM (*p = 0.129*) (Table 2).

These data show that the hyperintense PML rim is a heterogeneous region, with a combination of components with reduced and increased diffusion on both b1000 and b3000. Moreover, delineation of the lesion rim with b3000 results in smaller areas, excluding some components with increased diffusion.

## Discussion

The American Academy of Neurology (AAN) Neuro-infectious Disease Section recently stated that *compatible imaging findings* are one of the criteria required to establish a non-invasive PML diagnosis [11]. However, subcortical white matter T2-hyperintense and T1-hypointense signal alterations are rather non-specific. Also post-contrast enhancement is of little help, as it is present in other inflammatory and often concomitant white matter diseases. On the other hand, an asymmetrical ill-defined hyperintense DWI rim surrounding a hypointense core is a typical finding of full-blown PML lesions, and, when detectable, allows a more confident interpretation of otherwise nonspecific findings [18, 30].

In this study we found that b3000 improved the identification of the typical PML rim-and-core pattern by increasing the contrast between rim, core and NAWM, with a particularly evident signal drop within the core. The improvement was conspicuous despite the expected loss of signal and consequent lower SNR observed at high b-values [31, 32]. This is an important finding, suggesting that the use of b3000 may ultimately result in a higher number of patients meeting the criteria for non-invasive diagnosis of PML. This could potentially allow a higher number of patients to be diagnosed with PML without necessarily requiring invasive procedures, such as brain biopsy or JCV-positive CSF. In selected cases it may also suggest the diagnosis of PML despite a negative CSF, given the fact the JCV may initially be absent in the CSF in a small percentage of PML cases.

The usefulness of DWI in the setting of PML is, however, not only limited to diagnosis, as DWI can provide information about brain structure and disease-related changes. High signal regions on trace-DWI in PML lesions have been associated with the advancing edge of oligodendrocyte infection [22], whereas the hypointense core of older and larger lesions was assumed to represent the irreversibly damaged brain tissue with cellular debris [33]. Indeed, the role of PML DWI core as a marker of disease-associated damage was confirmed by Cosottini et al. [25], who found a significant correlation of disease duration and severity with the core volume.

In our sample the PML core showed high ADC values in both b1000 and b3000, consistently with an area of brain tissue destruction and in agreement with other authors’ reports [22, 25]. The PML rim showed average ADC values that were slightly higher than NAWM in both b1000 and b3000, signifying the bright DWI rim at least partly results from a T2 shine-through effect.

Despite average increased ADC values within the rim, in our sample this was often heterogeneous, with some foci of low ADC values in both b1000 and b3000, in agreement with other reports [22, 23]. Of note, b3000 and b1000 DWI similarly depicted the volume of PML lesions, but differed in the definition of the rim and core subsets, with a smaller and more stringent delineation of the rim in favour of a larger core volume with b3000. In particular, b3000 DWI was found to exclude some areas with normal to high ADC values from the rim, in keeping with an increased diffusion-weighting power with high b-values. This also resulted in average ADC values of the rim being similar to the NAWM on b3000, instead of slightly higher than NAWM as on b1000. It is thus logical to surmise that those regions that were reclassified by b3000 from rim into core correspond to those with the highest ADC within the rim on b1000 imaging, representing areas with a relatively more advanced infection stage with cytolysis and debris accumulation. Therefore, b3000 DWI can reduce the heterogeneity of the rim by retaining only the areas with the highest diffusion-weighted contribution and thus with increased probability of active demyelination.

Identifying the portion of the rim expressing active demyelination, potentially responding to therapeutic measures, from already damaged tissue is crucial information for clinical decisions and for prognostic stratification. To prove conclusively that the b3000 is more accurate than b1000 in detecting a rim with active demyelination, one would require histology from multiple biopsy sites of the rim, which is not part of the current clinical practice in the management of PML. Nevertheless, based on our data, it appears not unreasonable to conjecture that DWI with a high b-value may provide more precise biomarkers of the areas with infectious activity (i.e. the lesion rim) and irreversible brain damage (i.e. the lesion core).

The main limitations of this work are the retrospective nature and the limited number of patients analysed. The sample size is relatively small due to the relatively rarity of PML even in large tertiary referral centres, but comparable to previous studies on this topic (less than ten patients per study) [25, 33, 34]. In this initial study with proof-of-concept purposes we used consensus between two observers to outline the rim and core, but future larger studies would certainly benefit from intra- and interobserver variability assessment. Due to scanner variation and software upgrades over time, there were some inevitable variations in acquisition parameters between patients. To overcome this limitation we decided to analyse the data by using relative measures (SIRs) within each patient, as this approach minimises the effect of parameter variations between patients.

This study was conducted in HIV patients who were clinically symptomatic and therefore had relatively sizeable PML lesions. Future studies may need to look at the usefulness of high b-value DWI for early PML detection in other patient cohorts at risk, such as MS patients on natalizumab undergoing routine surveillance imaging who are asymptomatic and usually have smaller lesions [7, 8].

## Conclusions

Our study shows that DWI with a b-value of 3,000 s/mm^2^ allows a more conspicuous depiction of the typical pattern of PML, potentially increasing MRI accuracy for non-invasive PML diagnosis. Moreover, we showed that the DWI high-signal rim is heterogeneous being composed of regions with different ADC values, and that b3000 displays a thinner rim than b1000, excluding some areas with high ADC values, which are reclassified into the core. Given the prognostic relevance of the rim and core as a biomarker of disease activity and of tissue damage, respectively, b3000 DWI may well be potentially useful to assess treatment response, and to predict clinical outcomes in PML, which will need to be further investigated in future prospective trials.

## Electronic supplementary material

Below is the link to the electronic supplementary material.

ESM 1 (GIF 34 kb)

High Resolution Image (TIF 9944 kb)

## Abbreviations

ADC: Apparent diffusion coefficient
B1000: b-value of 1,000 s/mm^2^
B3000: b-value of 3,000 s/mm^2^
cART: Combined anti-retroviral therapy
CSF: Cerebrospinal fluid
DWI: Diffusion-weighted imaging
HIV: Human immunodeficiency virus
JCV: JC Virus
MS: Multiple sclerosis
NAWM: Normal-appearing white matter
PML: Progressive multifocal leukoencephalopathy
