# Supplementary figures and images for: High b-value diffusion-weighted imaging in progressive multifocal leukoencephalopathy in HIV patients
Source: Eur Radiol. 2017 Feb 6;27(9):3593–9. doi: 10.1007/s00330-017-4761-8 (PMC5544784; doi:10.1007/s00330-017-4761-8)

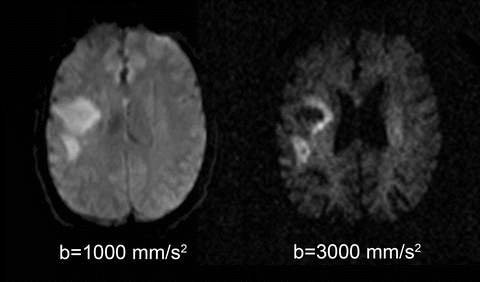

Supplement: Supplementary file 1 — (GIF 34 kb) [file 330_2017_4761_Fig4_ESM.gif]

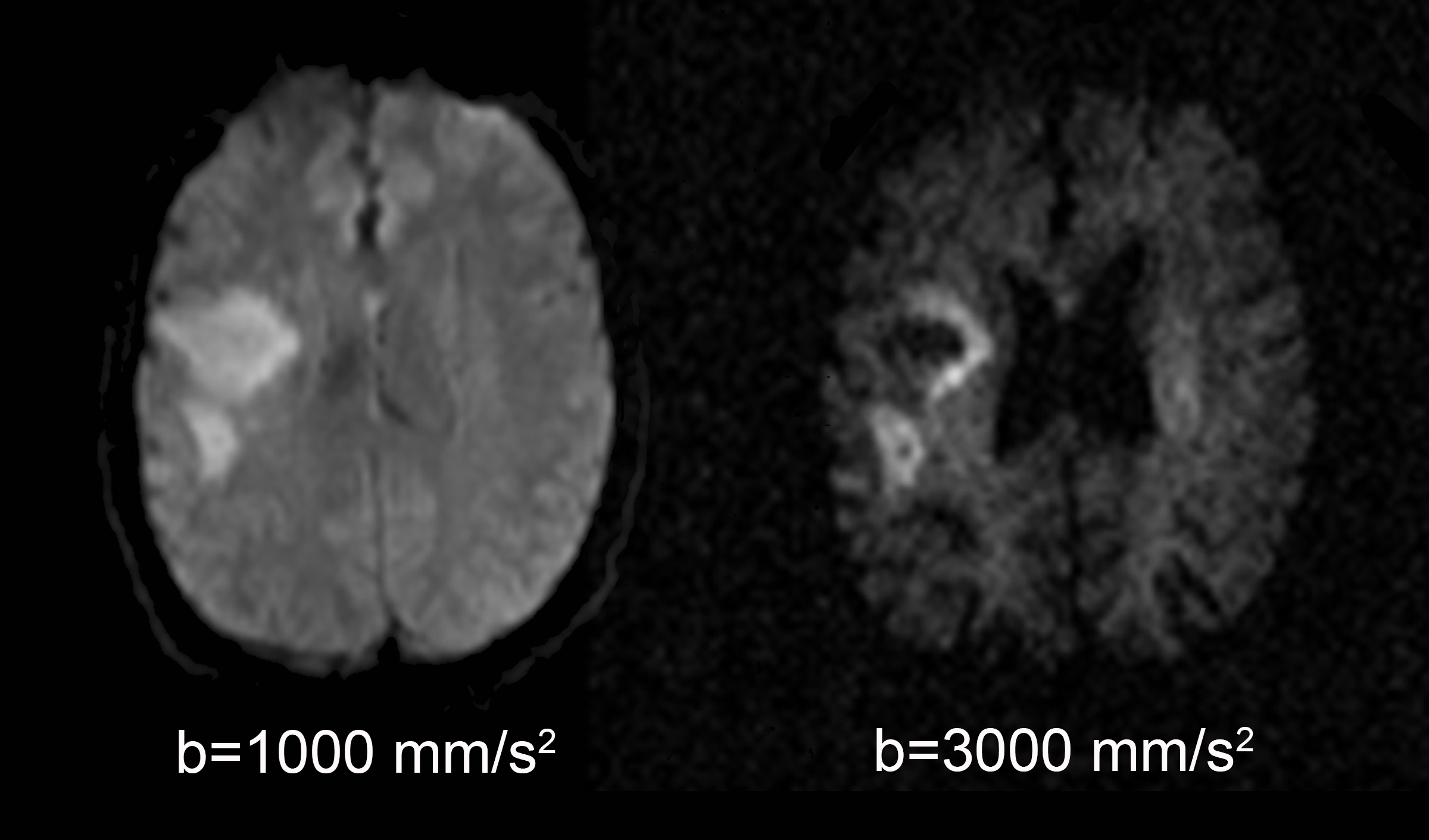

Supplement: Supplementary file 2 — High Resolution Image (TIF 9944 kb) [file 330_2017_4761_MOESM1_ESM.tif]
